# Dynamic Range Compression in the Honey Bee Auditory System toward Waggle Dance Sounds

**DOI:** 10.1371/journal.pone.0000234

**Published:** 2007-02-21

**Authors:** Seiya Tsujiuchi, Elena Sivan-Loukianova, Daniel F. Eberl, Yasuo Kitagawa, Tatsuhiko Kadowaki

**Affiliations:** 1 Graduate School of Bioagricultural Sciences, Nagoya University, Chikusa, Nagoya, Japan; 2 Department of Biological Sciences, University of Iowa, Iowa City, Iowa, United States of America; Centre de Recherches su la Cognition Animale - Centre National de la Recherche Scientifique and Université Paul Sabatier, France

## Abstract

Honey bee foragers use a “waggle dance” to inform nestmates about direction and distance to locations of attractive food. The sound and air flows generated by dancer's wing and abdominal vibrations have been implicated as important cues, but the decoding mechanisms for these dance messages are poorly understood. To understand the neural mechanisms of honey bee dance communication, we analyzed the anatomy of antenna and Johnston's organ (JO) in the pedicel of the antenna, as well as the mechanical and neural response characteristics of antenna and JO to acoustic stimuli, respectively. The honey bee JO consists of about 300–320 scolopidia connected with about 48 cuticular “knobs” around the circumference of the pedicel. Each scolopidium contains bipolar sensory neurons with both type I and II cilia. The mechanical sensitivities of the antennal flagellum are specifically high in response to low but not high intensity stimuli of 265–350 Hz frequencies. The structural characteristics of antenna but not JO neurons seem to be responsible for the non-linear responses of the flagellum in contrast to mosquito and fruit fly. The honey bee flagellum is a sensitive movement detector responding to 20 nm tip displacement, which is comparable to female mosquito. Furthermore, the JO neurons have the ability to preserve both frequency and temporal information of acoustic stimuli including the “waggle dance” sound. Intriguingly, the response of JO neurons was found to be age-dependent, demonstrating that the dance communication is only possible between aged foragers. These results suggest that the matured honey bee antennae and JO neurons are best tuned to detect 250–300 Hz sound generated during “waggle dance” from the distance in a dark hive, and that sufficient responses of the JO neurons are obtained by reducing the mechanical sensitivity of the flagellum in a near-field of dancer. This nonlinear effect brings about dynamic range compression in the honey bee auditory system.

## Introduction

The honey bee *(Apis mellifera)* uses various chemical and physical stimuli for communication. One of the best-characterized forms of honey bee communication is the forager's “waggle dance”, which informs nestmates about the direction and distance to locations of attractive food [1]. This dance consists of a series of alternating left-hand and right-hand loops, interspersed by a phase in which a dancer waggles her abdomen. The duration of the waggle run represents the distance to the food location. The direction of the waggle run relative to gravity corresponds to the direction with respect to the sun's azimuth. During the waggle run, the dancer waggles her abdomen while vibrating her wings, thereby generating various sounds and air flows. In addition, other signals such as temperature [2], odor [1], tactile contact [3], and comb vibration [4] have been suggested to assist followers to find, orient towards, and follow the waggle dancer. Airborne signals emitted by the dancer have been extensively studied. They consist of roughly 30 pulses per second, each pulse with a duration of about 20 ms and a carrier frequency of 265 Hz, air flows of a carrier frequency of 12–15 Hz, and jet flows [5]–[8]. Behavioral experiments demonstrated that honey bees can hear near-field sounds by detecting air-particle movements with Johnston's organ (JO) located at the second segment (pedicel) of the antenna [9]–[11]. This hearing mechanism is ideal for the followers located only millimeters away from a dancer. Furthermore, these experiments suggested that honey bees can learn, and discriminate between, different sound frequencies. JO is an antennal chordotonal organ, specialized for hearing in some insects, as best characterized in two Dipterans, mosquito and *Drosophila melanogaster* [12]. It consists of hundreds to thousands of scolopidial units, each composed of 2–3 neurons and several support cells [13]–[15]. JO transduces the mechanical vibration of the flagellum (the third antennal segment) into the electrophysiological activation of chordotonal neurons. These neurons project to the antennal mechanosensory region of the brain for further auditory processing.

Recent studies have shown that honey bees estimate the distance flown by optic flow and translates it to the duration of the “waggle dance” [16], [17]. The followers then need to perceive the duration of waggle run and translate it to the distance they are expected to fly. These results thus suggest neural templates for the amount of image motion and time period in the honey bee brain. To understand the mechanism of honey bee dance communication from detection to interpretation of dance messages at the neuronal level, we first started to characterize the anatomy and mechanical response characteristics of the antenna, and electrophysiological recordings of the antennal nerve in response to various near-field acoustic stimuli.

## Materials and Methods

### Morphological analysis of honey bee antennae by scanning electron microscopy

The antennae were removed from bees with scissors, immediately mounted on double-stick tape, and observed with a S-3000N scanning electron microscope (Hitachi) at several different magnifications with an acceleration voltage at 10.0 kV.

### Ultrastructural analysis of honey bee JO by transmission electron microscopy

The antennae were removed from bees as above, and the most of distal flagellum segments were removed to facilitate infiltration. They were then fixed by immersion overnight at 4°C in a fixative containing 2.5% glutaraldehyde, 2.0% paraformaldehyde, and 0.04% CaCl_2_ in 0.1M phosphate buffer (PB) at pH 7.4. The antennae were washed in PB, post-fixed with OsO_4_, dehydrated in an ethanol series, and embedded in Polybed 812. Ultrathin sections (75 nm) were stained with aqueous uranyl acetate and lead citrate, and were examined with a Hitachi 7000 electron microscope.

### Electrophysiological recordings of JO responses

Recordings of honey bee sound-evoked potentials (SEPs) were performed with pollen-foragers of unknown age unless otherwise noted. Honey bees with known age were prepared by collecting newly emerged bees from combs isolated from a hive, and putting the bees back to the hive after marking their thoraxes. Recordings for each stimulus and age variation were taken from at least 5 animals. For recording, bees were anesthetized by brief chilling on ice, and introduced into trimmed 1ml micropipette tips to expose the front of the head and antennae. The micropipette tip was mounted on a movable stage, and only bees with stabilized heads were used for recording. Tungsten electrodes were electrolytically sharpened, and one electrode was inserted into the joint between the first and second antennal segment while the other was simultaneously inserted into the head capsule. For lesions of the JO, the pedicel was stabbed by a tungsten needle several times before recording. To examine the effect of the flagellum length on JO responses, the flagellum was removed with scissors from the distal end before recording. The unfiltered differential AC signal was amplified 1000-fold by a DAM-50 amplifier (WPI) and sent to an InstruNet Model 100B acquisition board (GWI) in a Macintosh PC. The sampled signals were analyzed with Super Scope II software (GWI). To produce frequency spectra, the averaged responses to 265 and 750 Hz sine stimuli were collected and subjected to the FFT with a rectangular window. The response magnitudes were then calculated as path lengths at the corresponding frequency.

Stimulus traces were generated with Super Scope II at 13.3 kHz. The pulse stimulus trace consisted of 5 pulses (5 msec/pulse) with 500 Hz carrier frequency at 35 msec intervals, with the first pulse initiating at 15 msec. The “waggle dance” stimulus trace consisted of 4 pulses (20 msec/pulse) of a 265 Hz sine wave at 34 msec intervals, with the first pulse initiating at 21 msec. The sine wave traces were 100 msec tone bursts with linear on- and off-ramps. The computer-generated signals were amplified with a stereo amplifier A-D1 (Pioneer) and a 14 cm 5 Ohm 25 W speaker (Onkyo). The sound was delivered frontally to the bee's antenna through 0.8 cm (i. d.) Tygon tubing with one end connected via a funnel-shaped adaptor to the speaker. A 1 ml micropipette tip, cut to a 7 mm circular opening and plugged loosely with cotton to reduce echo, was inserted into the other end of the tubing and mounted close to the bee; antennal flagellums were kept within the hemisphere (3.5 mm) circumscribed by the tip opening to maintain near-field acoustic conditions [18].

### Simultaneous measurements of antennal vibrations and acoustic parameters of sound stimuli

Bees were introduced into the trimmed 1ml micropipette tips and mounted as above except the head/scape (the first antennal segment) and scape/pedicel joints of antenna were fixed with superglue to prevent the antennal movement by the head and antennal muscles [19], [20]. Only one antenna per animal was examined. Measurements were made with 4 live and 2 dead animals (caused by abdominal injection of 70% ethanol immediately after measuring the *in vivo* response) using pure tone acoustic stimuli of various frequencies and intensities. A laser Doppler vibrometer, CLV-700 (Polytec) and a near-field scanning probe (Microflown Technologies) were used to simultaneously measure the vibration velocity of antenna and the particle velocity in the surrounding air, respectively. The antennae were positioned between the laser vibrometer and the delivery end of Tygon tube (7 mm circular opening) from the loudspeaker, all of which were linearly aligned. The acoustic stimuli were delivered perpendicular to the axis of the antenna, and thus laser measurements were made coaxially with the direction of antennal movements.

The Microflown scanning probe is a MEMS-based acoustic sensor directly measuring particle velocity instead of sound pressure, which is usually measured by conventional microphones. The micromachined sensor is based on two heated extremely thin wires. A particle velocity signal in the perpendicular direction of the wires changes the temperature distribution instantaneously, since the upstream wire is cooled more by the air flow than the downstream wire. The resulting resistance difference provides a broadband linear signal with a figure of eight directionality that is proportional to the particle velocity up to sound levels of 135 dB. Between 100 Hz and 1 kHz, frequency and phase response signal is flat. The scanning probe was positioned next to the antenna (distance 2 mm) with its 45 degree angled sensor head (width 2 mm) perpendicular to the direction of sound propagation. We measured the particle velocity at various positions away from the delivery end of sound source, and found that the signals are constant within about 7 mm from the end. The antennal vibrations in the presence and absence of the sensor were constant, suggesting that the sensor did not affect the acoustic field at the position of the antenna. In addition, the sensor signals in the presence and absence of the antenna were constant, suggesting that the antenna did not affect the acoustic field at the position of the sensor head.

The laser vibrometer was positioned at 31 cm, corresponding to the focal length of the laser optics, away from the antenna. The laser-beam spot position was set at the distal tip of the flagellum by video monitoring. The reflection properties of the antennae were sufficient to obtain highly coherent measurements (range 0.8∼0.9) at the sound frequencies applied to the antennae.

To analyze the data, the laser and probe signals were digitized and analyzed using Bruel & Kjaer 3560-L and 7700N2 PULSE Lite Software system. To produce frequency spectra, groups of 5 windows, each 100 msec in length, were collected, subjected to the FFT with a rectangular window, and subsequently averaged. The magnitude and phase responses of the antennal vibration velocity were then normalized to those of the scanning probe by computing transfer functions between the laser and the probe signal. The transfer functions were calculated as the cross spectrum of the laser and the probe signal divided by the auto spectrum of the latter. The noise level in each measurement was examined by computing the coherence function, and only highly coherent measurements (providing phase and amplitude information with minimal contamination from unrelated noise) were used for analysis.

## Results

### External morphology of the honey bee antenna

**Figure 1:**
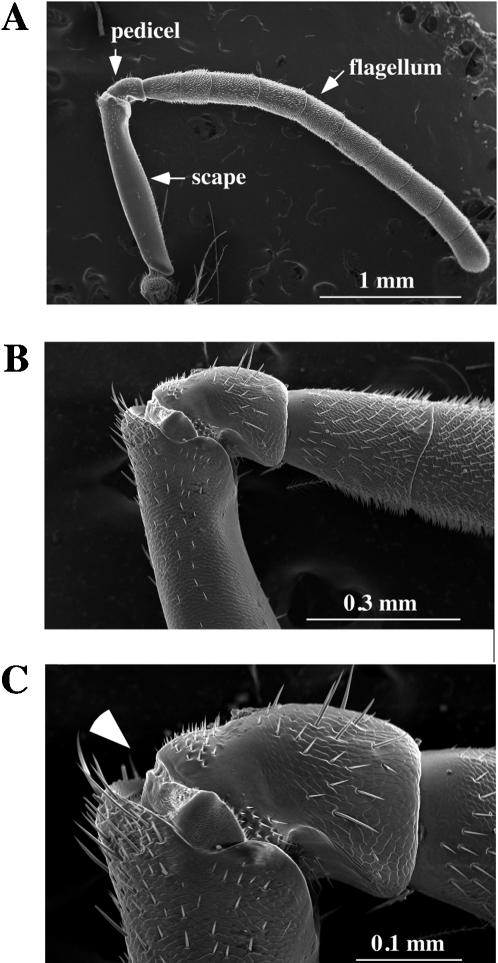
External morphology of the honey bee antenna. The honey bee antenna was examined by scanning electron microscopy with different magnifications (A; x40, B; x150, C; x 300). Two proximal antennal segments (scape and pedicel) and the ten segments of the flagellum are indicated by arrows in A. Arrowhead in C indicates the position of electrode insertion for SEP recordings. The scale of each panel is shown by a white bar.

Behavioral experiments suggested that honey bees detect near-field sound with JO in the pedicel of the antenna [11]. We thus examined the detailed morphology near the pedicel by scanning electron microscopy (SEM). The honey bee antenna is 4–5 mm long (Fig. 1). In contrast to the *Drosophila* antenna, which has a very short proximal scape, a rather round pedicel, and a pendulous funiculus with a fine perpendicular feathery arista [21], the honey bee antenna is more uniformly stout throughout its length and consists of a long proximal scape, followed by a more round pedicel, and capped with a regular series of ten flagellar segments (Fig. 1). The structure corresponding to the *Drosophila* arista, which imposes rotational forces on the third antennal segment, is not present in the honey bee. The joints at both ends of the pedicel are clearly defined (Fig. 1C), and the JO, the chordotonal organ that serves as the sensory element of the honey bee antennal movement, resides in the pedicel. We found that the flagellum can be moved at the joint between the pedicel and flagellum in response to acoustic stimuli (see below).

### The ultrastructure of honey bee JO

**Figure 2:**
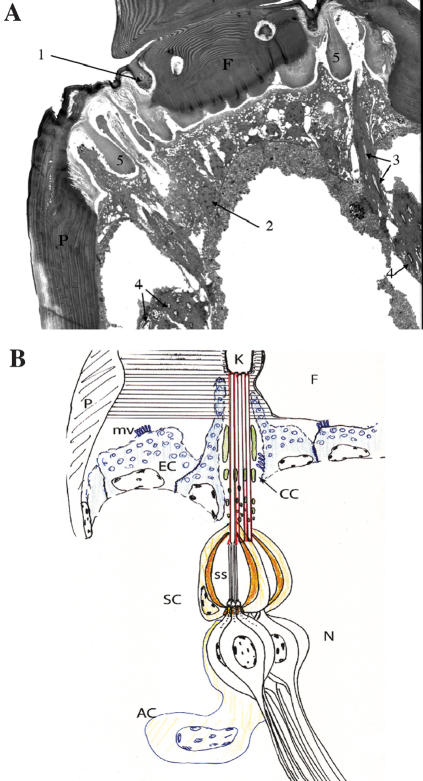
Organization of the honey bee JO. (A) TEM picture of the honey bee JO is shown. The morphology and positions of the cuticular knob (1), epithelial cell (2), quasi-longitudinal section of long sensory processes (3), quasi-transverse section of scolopidia (4), soft chitin (5), pedicel (P), and flagellum (F) are indicated. (B) The summary of the ultrastructural analysis of the honey bee JO is shown. At the joint of the pedicel (P) and flagellum (F), the cuticle is organized in a complex pattern of radial fibrils (horizontal lines), with circular fibrils surrounding the cuticular “knobs” (K) to which the scolopidia are attached. There are approximately 48 knobs evenly distributed around the circumference of the flagellum at its joint with the pedicel. Epithelial cells (EC; blue) exhibit extensive apical microvilli, likely for the copious secretion of cuticle proteins. The epithelial cell cytoplasm is filled with spongiform membranous organelles, likely also reflecting high secretion levels. Each cuticular knob is the attachment site of 3–10 scolopidia. Each scolopidium forms an independent dendritic cap (red), and these are surrounded by cap cells (CC) which enclose electron dense rods (green) that are thick apically but divide more basally into multiple finer rods. The scolopale cell (SC) of each scolopidium forms a spindle-shaped cage of scolopale rods (organe) and encloses an extracellular scolopale space (ss), through which the ciliary outer dendritic segments of three neurons (N) extend. Morphologically, honey bee JO scolopidia are amphinematic, containing cilia of both Types I and II (see classification described in ref. 30). The two Type I cilia are of uniform diameter, contain an axoneme along their entire length, and attach to the basal end of the dendritic cap; the single Type II cilium contains an axonemal segment up to the dendritic cap, then a wider non-axonemal segment with loose microtubules that continues throughout the length of the dendritic cap. Basally, there are accessory cells (AC) of uncertain classification. The structure of the honey bee JO is consistent with a sensory function for flagellar vibration.

The general structure of the honey bee JO has been described [22]. We analyzed the ultrastructure of the honey bee JO by transmission electron microscopy (E. Sivan-Loukianova, S. Tsujiuchi, T. Kadowaki, and D. F. Eberl; in preparation; Fig. 2). The cuticular arrangement of the pedicel-flagellum joint is rather elaborate, consistent with the need to support a flagellum of considerable mass while still allowing flexibility for acoustically induced vibrations. There are about 47–49 cuticular “knobs” around the circumference of the pedicel, each one connected to 3–10 scolopidia. Using an estimated average of 6.5 scolopidia per knob, there would be about 300–320 scolopidia in the honey bee JO. Thus, the number of sensory units is comparable to that of flies but less than mosquitoes, where there are 150–200 and 3000–7000 units in the *Drosophila* and mosquito JOs, respectively [13]–[15]. It is important to note that these sensory neurons do not directly attach with the flagellum.

### The flagellar vibrations induced by near-field acoustic stimuli

**Figure 3:**
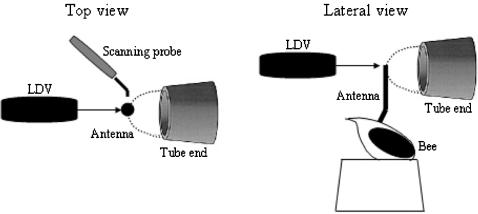
Simultaneous measurements of the flagellar vibration velocity and the particle velocity of the surrounding air. Experimental arrangement in top and lateral views is shown. The laser doppler vibrometer (LDV), the honey bee antenna, and the sound tube were linearly aligned along with the optical axis of the LDV as shown. The vibrometer was positioned at 31 cm away from the flagellum. Sound stimuli are delivered through a Tygon tube ending in a 7 mm opening close to the honey bee. The dashed line indicates the hemispherical zone where full near-field acoustic conditions are maintained. The scanning probe to measure the particle velocity of the surrounding air was positioned at 2 mm away from the flagellum. The probe head was aligned perpendicular to the direction of sound propagation. The joints between head and scape as well as scape and pedicel were fixed with glue to prevent the antennal movements by muscle. The spot position of laser beam was fixed at the tip of the flagellum. All items are not in scale.

**Figure 4:**
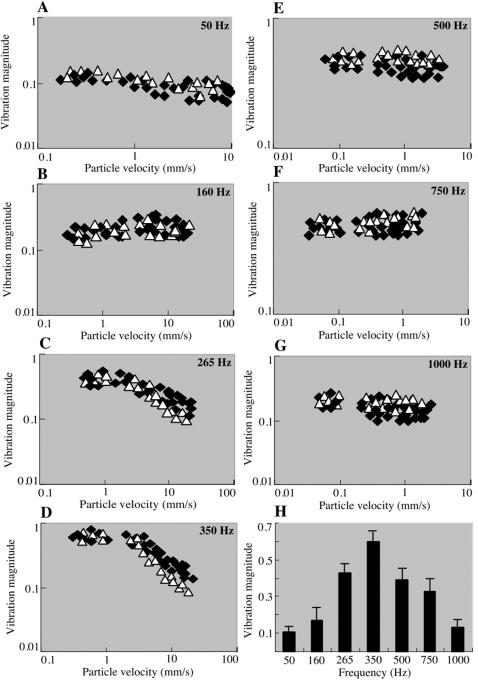
Intensity and frequency characteristics of honey bee flagellar vibrations in response to acoustic stimuli. The vibration (response) magnitudes, the ratio of the flagellar vibration velocity to the air particle velocity, of 4 live (solid square) and 2 dead (open triangle) bee flagellar tips to pure-tone stimulation at different frequencies (50–1000 Hz) are plotted against the particle velocity (A-G). The data points are parallel to the intensity axis at 50 (A), 160 (B), 500 (E), 750 (F), and 1000 (G) Hz, demonstrating that the flagellar vibration velocity linearly increases as a function of the air particle velocity. The response magnitudes to 265 (C) and 350 (D) Hz stimuli are non-linear, they show the maximum only at intensities between 0.3–4 mm/s, and then decrease at intensities above 4 mm/s. (H) Bar graph shows the mean response magnitudes to stimulation at different frequencies obtained with 0.3 mm/s air particle velocity. Error bars indicate standard error. The resonance frequency of the honey bee flagellum would be around 350 Hz.

**Figure 5:**
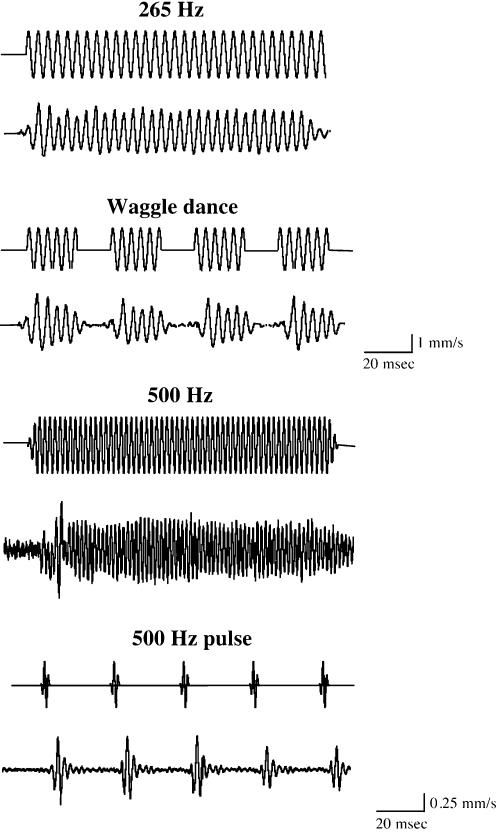
Temporal patterns of honey bee flagellar vibrations in response to continuous and pulse tone stimuli. The time traces of honey bee flagellar vibrations in response to the continuous and pulse tone stimuli at 265 and 500 Hz frequencies are shown. The 265 and 500 Hz pulse stimuli represent the honey bee “waggle dance” sound and *Drosophila* courtship song, respectively. For each measurement, upper and lower traces indicate the time trace of sound stimulus and the flagellar vibration velocity, respectively. Each vibration velocity shown is the averaged response of 10 trials in a single antenna. Note that the vertical scales for 265 and 500 Hz stimuli are different, 1 and 0.25 mm/s, respectively. The honey bee antennal flagellum has the ability to preserve both frequency and temporal patterns of acoustic stimuli applied.

Honey bee antennae are thought to be deflected by the bulk air movement in the acoustic near-field of a sound source [23]. If the model is correct that the vibration of the long flagellum relative to the pedicel causes the mechanical stretching and activation of sensory neurons in the JO, then it should be possible to detect these flagellar vibrations. We first designed stimuli with 100 msec tone bursts of various sinusoidal frequencies (50–1000 Hz), and also simulated the sound of the honey bee “waggle dance” with four 20 msec 265 Hz pulses, and the *Drosophila* pulse song with short 500 Hz pulses. To examine the mechanical response characteristics of honey bee antennal flagellum, we simultaneously measured the vibration velocity of the flagellum and the particle velocity of the surrounding air in response to pure tone stimuli of various frequencies (50–1000 Hz) and intensities (particle velocity 0.05–20 mm/s) as illustrated in Fig. 3. The vibration (response) magnitude (the relative vibration velocity; the vibration velocity of the flagellum/the air particle velocity) is plotted against the particle velocity at each stimulus frequency (Fig. 4A–G). If the vibration velocity is proportional to the particle velocity (a linear system), the data points of the magnitude should be parallel to the intensity axis. This is indeed the case for the carrier frequency of 50, 160, 500, 750, and 1000 Hz in the range of intensities tested. However, in response to 265 and 350 Hz stimuli, the maximum mechanical sensitivity is observed at low particle velocity between 0.3 and 4 mm/s, and decreases at the particle velocities above 4 mm/s. For example, in response to 350 Hz stimuli, the sensitivities are 0.6 and 0.2 at the particle velocities of 0.3 and 10 mm/s, respectively. We also analyzed the mechanical response characteristics of the post-mortem antennal flagellum immediately after measuring the *in vivo* response. The mechanical sensitivity of the post-mortem antennal flagellum (plotted as white triangles in Fig. 4A–G) is comparable to that of *in vivo* in terms of both magnitude and non-linearity. One exception is that the post-mortem sensitivities in response to 265 and 350 Hz stimuli are approximately half of those *in vivo* at the high particle velocities. This is probably because the post-mortem joint stiffness is higher than *in vivo*. The mechanical sensitivities obtained with the particle velocity of 0.3 mm/s at each stimulus frequency are shown in Fig. 4H. The highest sensitivity is observed at 350 Hz, suggesting that the resonance frequency of honey bee antennal flagellum would also be around 350 Hz. To determine whether the vibrations of the flagellum represented movement of the flagellum relative to the pedicel, we measured movements of the pedicel during acoustic stimulation and found no significant movement (data not shown). The fact that the pedicel remains effectively stationary confirms that the sound-evoked vibrations move the flagellum at its joint with the pedicel, and is consistent with mechanical actuation of the JO, as monitored by our ultrastructural analysis of the JO and electrophysiological experiments (see below) as well as acoustic behavioral experiments [11]. We also examined the mechanical response characteristics of the flagellum at the base, and found that they are identical to those measured at the tip except the decreased mechanical sensitivity (data not shown). The temporal patterns of the flagellar vibrations in response to continuous and pulse sound stimuli of carrier frequency 265 and 500 Hz are shown in Fig. 5. In response to the *Drosophila*-like train of five pulses and the honey bee “waggle dance” sound, the flagellar vibrations are reproducibly seen after each pulse with the same temporal patterns. Thus, the flagellar vibrations basically follow the frequency and temporal patterns of acoustic stimuli applied.

### Recording extracellular compound potentials originating from JO

**Figure 6:**
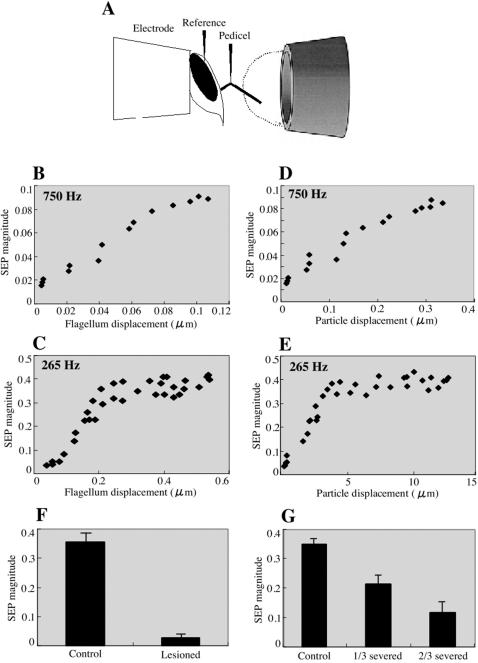
Intensity characteristics of SEPs in response to acoustic stimuli. (A) Schematic representation of the preparation is shown. Honey bee heads are fixed and exposed from the end of a micropipette tip, and the recording electrode is inserted at the joint between the scape and pedicel (Fig. 1C) and the reference electrode penetrates the head cuticle. Sound stimuli are delivered through a Tygon tube ending in a 7 mm opening close to the honey bee. The dashed line indicates the hemispherical zone where full near-field acoustic conditions are maintained. The SEP magnitudes in response to 750 (B and D) and 265 (C and E) Hz tone stimuli are plotted against the flagellum (B and C) and air particle (D and E) displacements. The SEP magnitudes linearly increase with the flagellar tip displacement up to 100 nm. The response of JO neurons can be detected by 20 nm displacement of flagellar tip in response to 750 Hz stimuli. The background SEP magnitude is <0.02. The response of JO neurons saturates by the flagellar tip displacement above 200nm in response to 265 Hz stimuli. (F) Bar graph shows the mean SEP magnitudes in response to 265 Hz acoustic stimuli at 7 mm/s air particle velocity. SEPs were measured before (Control) and after (Lesioned) lesions in the pedicel. Error bars indicate standard error (N = 5 antennae per treatment, where the SEP of each antenna is the averaged response to 10 trials). (G) SEPs were measured before (Control) and after cutting off the distal one-third (1/3 severed) and two-thirds (2/3 severed) segment of the flagellum. Bar graph of the mean SEP magnitudes as above is shown. Error bars indicate standard error.

To measure the electrophysiological response of the JO, we recorded sound-evoked compound potentials (SEP) from the antennal nerves of intact honey bees. Extracellular potentials were recorded with an electrode inserted at the joint between the scape and pedicel (Fig. 1C and Fig. 6A). At this point, the antennal nerve includes axons projecting from the chordotonal organs and other sensory organs in the more distal segments. Another electrode was inserted into the head cuticle as a reference.

First, the transformation of the flagellar vibrations to the JO neuronal activity was quantified. We analyzed the relationship between the flagellar tip displacement and the magnitude of SEP in response to 750 and 265 Hz pure tone as shown in Fig. 6B–E. The flagellar vibration velocity was converted to the corresponding displacement by the equation, displacement = velocity/2πf. In response to 750 Hz stimuli, the SEP magnitude is positively correlated with the flagellar tip displacement up to 100 nm (Fig. 6B). At higher displacements above 200 nm in response to 265 Hz stimuli (achieved by air particle displacements above 5 µm, Fig. 6E), the SEP magnitude saturates (Fig. 6C). The minimum response of JO neurons can be detected by 20 nm displacement of the flagellar tip (achieved by 60 nm air particle displacement, Fig. 6D). To test whether the SEPs are derived from the JO neurons, we recorded the SEP by 265 Hz pure tone stimuli before and after lesion in the pedicel. As shown in Fig. 6F, the SEPs are no longer detectable after lesion, demonstrating that the SEPs are attributable to JO. Furthermore, the SEP magnitudes are dependent on the flagellum length as shown in Fig. 6G. This suggests that the mechanical forces exerted on the JO by the flagellar vibrations are directly translated into the SEP magnitudes.

**Figure 7:**
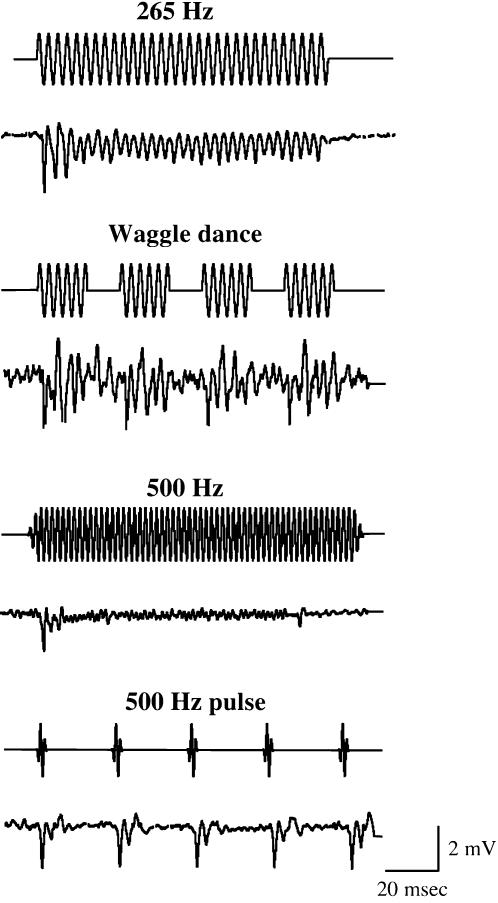
Temporal patterns of honey bee SEPs in response to continuous and pulse tone stimuli. The time traces of honey bee SEPs in response to the continuous and pulse tone stimuli at 265 and 500 Hz frequencies are shown. The applied stimuli are the same as in Fig. 5. For each measurement, upper and lower traces indicate the time trace of sound stimulus and SEP, respectively. Each SEP shown is the averaged response of 10 trials in a single antenna. Both frequency and temporal patterns of SEPs are similar to those of the flagellar vibrations shown in Fig. 5.

The temporal patterns of the JO neural responses against continuous and pulse sound stimuli of carrier frequency 265 and 500 Hz are shown in Fig. 7. In response to the *Drosophila*-like train of five pulses and the honey bee “waggle dance” sound, the SEPs are reproducibly seen after each pulse with the same temporal patterns. The JO neural responses are robustly consistent with the flagellar vibration patterns (see Fig. 5).

### The response of JO neurons is dependent on the age of honey bee workers

**Figure 8:**
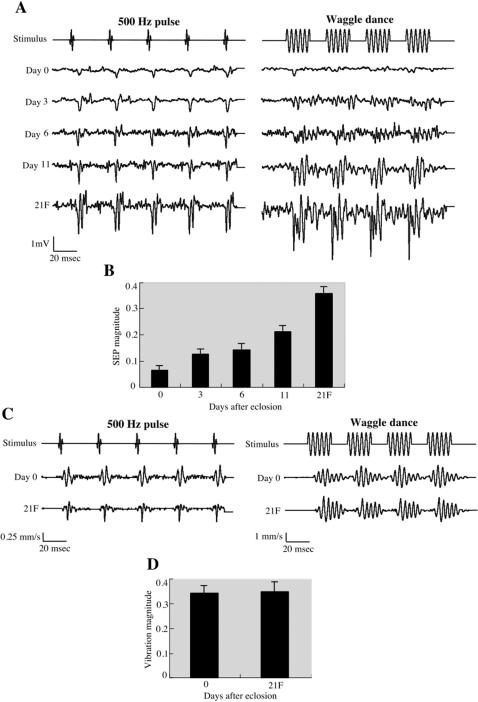
The age-dependent response of honey bee JO neurons to acoustic stimuli. (A) Representative time traces of SEPs of 0, 3, 6, 11 and 21 day old bees (the 21 day old bees are foragers (21F)) in response to 500 Hz pulse and “waggle dance” sound stimuli are shown. Each SEP is the averaged response of 10 trials in a single antenna. Note the vertical scale is 1 mV. (B) Bar graph of 10-trial SEP magnitudes obtained with 265 Hz continuous sound stimuli at 7 mm/s air particle velocity for N = 5 antennae per treatment. Error bars indicate standard error. (C) The time traces of flagellar vibrations of 0 day old bees (Day 0) and 21 day old foragers (21F) in response to 500 Hz pulse and “waggle dance” sound stimuli are shown. Each vibration velocity shown is the averaged response of 10 trials in a single antenna. The vertical scales of the velocity to 500 Hz pulse and “waggle dance” sound stimuli are 0.25 and 1 mm/s, respectively. (D) Bar graph shows the mean vibration magnitudes in response to 265 Hz continuous sound stimuli at 7 mm/s air particle velocity. Error bars indicate standard error.

Honey bee workers start foraging at approximately 3 weeks after eclosion under normal conditions [24]. Thus, they are thought to commence the dance communication at this age. We therefore tested whether the response of JO neurons to acoustic stimuli is dependent on the age of worker bees (Fig. 8A and B). In response to either 500 Hz pulse sound or “waggle dance” sound, the SEP of newly eclosed (0 day old) bees exhibits the lowest amplitude and slowest kinetics. The SEP magnitudes against the “waggle dance” sound increase and the kinetics of the extracellular potential fluctuations become faster by age. Because the response of JO neurons is dependent on the flagellar vibration (Fig. 6), we compared the flagellar vibrations of 0 day old bees and 21 day old foragers. As shown in Fig. 8C and D, the flagellar vibrations to 500 Hz pulse and “waggle dance” sounds are comparable between 0 day old bees and 21 days old foragers. These results suggest that the sensitivity of honey bee JO neurons to flagellar vibrations matures with age.

## Discussion

### Mechanical and response characteristics of honey bee antenna and JO neurons against acoustic stimuli

Honey bee antennae and JO were suggested to be air particle movement detectors for decoding the dance messages [6]. Furthermore, behavioral experiments suggested that honey bees hear by detecting air-particle movements with the JO [9]–[11]. We have therefore investigated the mechanical and neuronal response characteristics of honey bee antenna and JO against acoustic stimuli. We detected stimulus-specific fluctuations in the extracellularly recorded summated neuronal responses from the electrode inserted just proximal to the flagellum-pedicel joint, i.e. just proximal to where the JO is located. The sound-evoked vibrations of the flagellum at this joint and the relative position of the recording electrode strongly support the idea that SEPs are derived from the sensory neurons of the approximately 300 JO scolopidia. In fact, lesions in the pedicel abolished the SEPs (Fig. 6F). Thus, it is likely that the observed SEP is the aggregate extracellular product of many or all of these units. Moreover, the SEP magnitudes are dependent on the flagellum length (Fig. 6G), suggesting that the mechanical forces exerted on the JO by the flagellar vibrations are directly translated into the SEP magnitudes. Intriguingly, the frequency and temporal patterns of the acoustic stimuli are clearly resolved at the level of both flagellar vibrations and JO neuronal responses in honey bee (Fig. 5 and 7). Thus, the flagellar vibration patterns are faithfully translated into the JO neuronal responses which honey bees may use to detect and discriminate various acoustic stimuli of different frequencies and temporal patterns.

In contrast to the rotational activation of JO mediated by the laterally extending arista in the *Drosophila* antenna [21], [25], the honey bee antenna is activated by bending movements of the flagellum along, rather than around, the longitudinal axis of the antenna. This arrangement is more similar to that of the mosquito, with its long flagellar segments (see for example Fig. 1 in ref. 26). The large size of the bee antenna relative to those of *Drosophila* and mosquitoes may explain why much higher stimulus amplitudes are necessary for the honey bee antenna to vibrate and elicit the SEP compared to *Drosophila* and mosquito. For example, the maximum mechanical sensitivity of honey bee antenna is 0.6 (Fig. 4), in contrast to 4.3 for mosquito [26] and 1.3 for *Drosophila* [25]. Thus, the vibration velocity of the honey bee antenna never exceeds the air particle velocity. This is nevertheless appropriate in the respective behavioral contexts for these species, where the honey bee is able to use much more energy in the production of air flow by wing and abdominal vibrations. In fact, the air particle velocity of 250–300 Hz sound generated by wing vibration of dancer is 0.4–0.5 m/s at 2 mm away from dancer's abdomen in the perpendicular direction to the plane of the wings [6], [8]. Because the SEP of honey bee JO neurons reaches to the saturation at the air particle velocity above 7 mm/s at 265 Hz (Fig. 6E), the follower bees should be able to detect 265 Hz sound when their antennae are closed to the dancer's wings. The dancers also produce other air-borne signals during the “waggle dance”, 12–15 Hz and jet air flows generated by abdominal vibration and wing vibration, respectively [8]. The intensities of these air flows [8] are also sufficient to produce 5 µm air particle displacement, the saturation threshold of JO SEP response (Fig. 6E). These results thus suggest that the antennal vibrations induced by various air flows generated during “waggle dance” result in the maximum response of JO neurons which followers can use to decode the dance messages. The honey bee Johnston's organ containing approximately 1000 ciliated sensory neurons can sense 20 nm flagellar tip deflection caused by 60 nm air particle displacement. This is comparable to female mosquitoes except they require only 2–4 nm air particle displacement, presumably due to the high mechanical sensitivity [27]. Nevertheless, the honey bee antennal flagellum functions as one of the most sensitive (nanometer-range) movement detectors in insects.

The resonance frequency of honey bee antenna would be around 350 Hz when the intensity of acoustic stimuli is low (particle velocity <4 mm/s at 265–350 Hz). Because of non-linearity between the antennal deflection and the air particle displacement in response to intense stimuli of these carrier frequencies, the antennal resonance may shift toward higher frequency as observed in *Drosophila* [25]. The mechanical response characteristics of honey bee antenna *in vivo* and post-mortem against acoustic stimuli are comparable, suggesting that the JO neurons do not contribute energy to amplify the vibrations of the flagellum in contrast to *Drosophila* [28] and mosquito [26]. This is consistent with that the honey bee JO neurons connect with the cuticular “knobs” around the circumference of the pedicel, and do not directly associate with the flagellum (Fig. 2). Moreover, the total number of honey bee JO neurons (approximately 1000) would not be sufficient to support the motion of the large flagellum relative to mosquito and *Drosophila*. The mechanical response parameters of honey bee antenna to acoustic stimuli seem therefore primarily to be determined by the structural properties. The structural characteristics underlying the frequency and intensity specific amplification of the flagellar deflection remain to be determined.

The specific amplification of the flagellar vibration in response to weak acoustic stimuli of 265–350 Hz frequencies may allow honey bees to find the dancer generating 250–300 Hz sound from the distance in a dark hive. When the followers approach close to the dancer generating intense sound (see above), the antennal mechanical sensitivities are reduced so that the strength of mechanical forces exerted on JO could be adjusted to extend the dynamic range to support appropriate responses of JO neurons even at high stimulus intensity. Because the honey bee antenna and JO neurons have the nanometer-range sensitivity and the ability to preserve both frequency and temporal patterns of acoustic stimuli, honey bees may be able to detect and discriminate various air borne signals other than “waggle dance”.

### Age-dependent maturation of honey bee hearing

Honey bee workers perform a variety of tasks depending on their ages (age polyethism). Young workers feed and care for larvae and the queen, middle-age workers maintain the hive and store food, and older workers forage for nectar and pollen outside the hive [24]. As shown in Fig. 8, we have found that honey bee hearing appears to mature with age. Since the flagellar vibrations are comparable between newly emerged bees and older foragers, the smaller, broader peaks in the SEPs of young bees suggest that the JO is not fully functional at early adult stages. This contrasts with *Drosophila* in which newly eclosed flies show a strong auditory response [21]. There could be a physiological maturation in the sensory transduction mechanism, whether in the number or location of transduction channels, or regulation of their downstream electrochemical events. Age dependent maturation of JO neurons suggests that only honey bee workers old enough to forage can efficiently detect the dance language presented by other foragers. Meanwhile, other adult senses, for example, olfaction, should be fully functional at an early adult stage. These results together with our previous observation on the age and light dependent maturation of visual activity [29] demonstrate that the functional maturation of sensory systems is adjusted to become available just in time for the honey bee's need for particular senses during its life span.

Honey bees need to recognize the duration of waggle run and translate it into the expected amount of optic flow to determine their flight distance [16], [17]. These decoding processes of the dance message must be conducted in the brain by transferring the response information of JO neurons. The brain areas, neuronal circuits, and neurons responsible for decoding the dance language remain to be established. Biomechanical and electrophysiological characterization of honey bee antenna and JO neurons reported here will be valuable to give insight into these issues.
